# Exploring Antimicrobial Features for New Imidazo[4,5-b]pyridine Derivatives Based on Experimental and Theoretical Study

**DOI:** 10.3390/molecules28073197

**Published:** 2023-04-04

**Authors:** Mohammed-yassin Hjouji, Ahmed M. Almehdi, Hicham Elmsellem, Yousra Seqqat, Younes Ouzidan, Mohamed Tebbaa, Noura Ait Lfakir, Youssef Kandri Rodi, Fouad Ouazzani Chahdi, Marwa Chraibi, Kawtar Fikri Benbrahim, Mohamed A. Al-Omar, Abdulrahman A. Almehizia, Ahmed M. Naglah, Shaima A. El-Mowafi, Ahmed A. Elhenawy

**Affiliations:** 1 Laboratory of Applied Organic Chemistry, Faculty of Science and Technology Saiss, Sidi Mohammed Ben Abdallah University, Fez 30050, Morocco; 2 Department of Chemistry, College of Sciences, University of Sharjah, Sharjah P.O. Box 27272, United Arab Emirates; 3 Laboratory of Applied Chemistry and Environment (LCAE), Sciences Faculty, Oujda 60000, Morocco; 4 Laboratoire de Chimie-Physique et Biotechnologie des Biomolécules et Matériaux, Faculté des Sciences et Techniques, Université Hassan II, BP 146, Mohammedia 28800, Morocco; 5 Laboratory of Microbial Biotechnology, Faculty of Science and Technology Saïss, Sidi Mohamed Ben Abdellah University, Fez 30050, Morocco; 6 Drug Exploration & Development Chair (DEDC), Department of Pharmaceutical Chemistry, College of Pharmacy, King Saud University, Riyadh 11451, Saudi Arabia; 7 Peptide Chemistry Department, Chemical Industries Research Institute, National Research Centre, Dokki, Cairo 12622, Egypt; 8 Chemistry Department, Faculty of Science, Al-Azhar University, Cairo 11884, Egypt; 9 Chemistry Department, Faculty of Science and Art, Albaha University, Albahah 65731, Saudi Arabia

**Keywords:** antimicrobial activity, imidazo[4,5-b]pyridine, DFT, N-alkylation, CTP

## Abstract

5-bromopyridine-2,3-diamine reacted with benzaldehyde to afford the corresponding 6-Bromo-2-phenyl-3H-imidazo[4,5-b]pyridine (**1**). The reaction of the latter compound (**1**) with a series of halogenated derivatives under conditions of phase transfer catalysis solid–liquid (CTP) allows the isolation of the expected regioisomers compounds (**2**–**8**). The alkylation reaction of (1) gives, each time, two regioisomers, N3 and N4; in the case of ethyl bromoactate, the reaction gives, at the same time, the three N1, N3 and N4 regioisomers. The structures of synthesized compounds were elucidated on the basis of different spectral data (^1^H NMR, ^13^C NMR), X-Ray diffraction and theoretical study using the DFT method, and confirmed for each compound. Hirshfeld surface analysis was used to determine the intermolecular interactions responsible for the stabilization of the molecule. Density functional theory was used to optimize the compounds, and the HOMO-LUMO energy gap was calculated, which was used to examine the inter/intra molecular charge transfer. The molecular electrostatic potential map was calculated to investigate the reactive sites that were present in the molecule. In order to determine the potential mode of interactions with DHFR active sites, the three N1, N3 and N4 regioisomers were further subjected to molecular docking study. The results confirmed that these analogs adopted numerous important interactions, with the amino acid of the enzyme being targeted. Thus, the most docking efficient molecules, **2** and **4**, were tested in vitro for their antibacterial activity against Gram-positive bacteria (*Bacillus cereus*) and Gram-negative bacteria (*Escherichia coli*). Gram-positive bacteria were more sensitive to the action of these compounds compared to the Gram-negative, which were much more resistant.

## 1. Introduction

Today, infectious diseases caused by bacteria, fungi, viruses and parasites remain a major threat to public health and a challenge to world’s scientific community [1]. Antibiotic substances are molecules derived from secondary metabolism which have been particularly studied because of their importance in human therapy [2]. Since their discovery by Fleming in 1928 [3], antibiotics have become indispensable to the current system of health, helping and supplementing the functioning of the immune system against pathogenic microbes. Since then, humankind has had historic success in controlling morbidity due to infectious diseases through antibiotic therapy. Following the abusive use of chemo-therapeutic agents, microorganisms, through their potential to mutate, generate resistance mechanisms to the known classes of antimicrobials [4]. This has become a serious problem in recent years [4], and presents a continuous clinical challenge. Thus, the pressing need for new effective classes of antimicrobials with new modes of action remains necessary and all possible strategies should be explored. However, strategies to address this challenge include designing improved versions of already known classes of antimicrobials or designing new classes of molecules based on the enormous potential of natural products.

**Figure 1:**
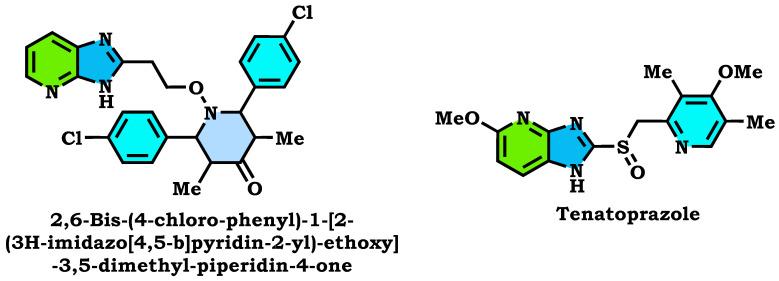
Structures of some drugs containing the imidazo[4,5-b]pyridine skeletons.

Pharmacological and therapeutic activities that present a variety of heterocyclic molecules containing an imidazo[4,5-b]pyridine pattern have greatly aroused the interest of researchers for the development of new routes to such compounds. They have often been defined as precursors in the synthesis of a variety of therapeutic agents. Indeed, they are endowed with anticancer [5,6,7,8], antimitotic [9] and tuberculostatic properties [10]. Recently, studies have shown that imidazo[4,5-b]pyridine derivatives can be evaluated as antagonists of various biological receptors AT_1_ and AT_2_, including angiotensin II [11] and thromboxane A_2_ [12]. Thus, some of those skeletons have, in particular, been introduced into structures of antibacterial agents [13] such as 2,6-Bis-(4-chloro-phenyl)-1-[2-(3H-imidazo[4,5-b]pyridin-2-yl)-ethoxy]-3,5-dimethyl-piperidin-4-one (Figure 1), a powerful antibacterial agent against Bacillus and *Staphylococcus aureus*. It also has antimycotic activity against *Aspergillus flavus* [14]. The best-known example of this family is tenatoprazole, used for the treatment of gastric and duodenal ulcers due to its inhibitory activity of the proton pump [15] (Figure 1).

Based on all the previous information, and in the interests of synthesizing new antimicrobial agents, we chose, in this work, to synthesize new imidazo[4,5-b]pyridine derivatives (**1**) on which we have introduced modifications using the N-alkylation reaction, under conditions of phase transfer catalysis solid–liquid [16] with a series of halogenated compounds such as Benzyl and ethyl ethanoate, known for their biological activities [17,18,19,20,21,22,23]. Theoretical calculations were performed by the DFT [24,25,26] to explain reasons that manage the alkylation reactions of imidazopyridine (**1**), and then synthesized compounds were characterized by spectroscopic techniques such as ^1^H NMR, ^13^C NMR and X-Ray diffraction. In addition, some of those compounds were evaluated for their antibacterial activities in vitro against *Escherichia coli* and *Bacillus cereus* bacteria.

## 2. Results and Discussion

### 2.1. Chemistry

**Scheme 1:**
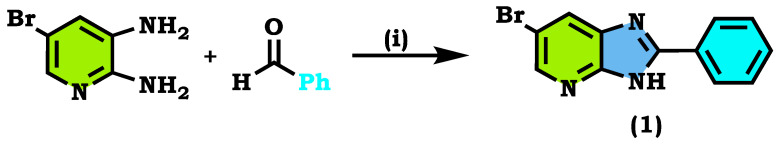
Synthetic route of 6-Bromo-2-phenyl-3H-imidazo[4,5-b]pyridine (**1**) and their conditions: (i) Reflux EtOH, I_2_, 24 h.

Among the derivatives of 2,3-diaminopyridine, 5-bromo-2,3-diaminopyridine appears as a potentially important synthon involved in the synthesis of imidazo[4,5-b]pyridine [27]. Indeed, the condensation of this compound with the benzaldehyde led to the formation of the expected imidazo[4,5-b]pyridine derivative (**1**) (Scheme 1).

**Scheme 2:**
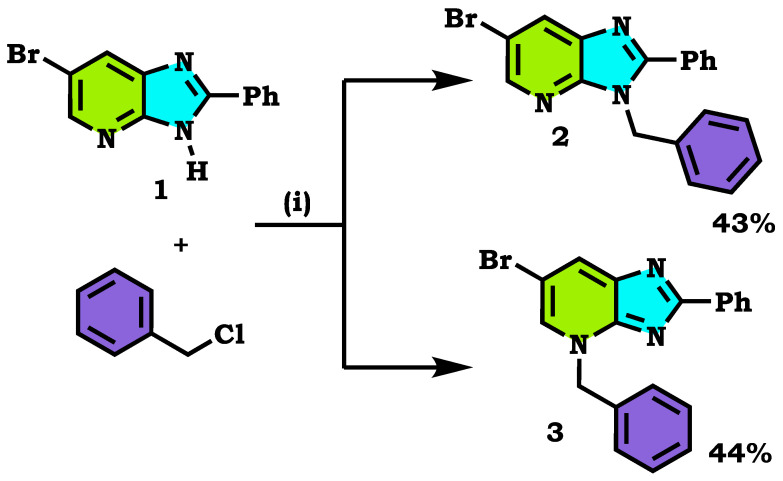
Synthetic route of novel imidazo[4,5-b]pyridine derivatives and conditions (**2**, **3**): (i) DMF (15 mL), K_2_CO_3_ (1.2 equiv), t-BAB (0.1 equiv), 6 h, RT.

**Scheme 3:**
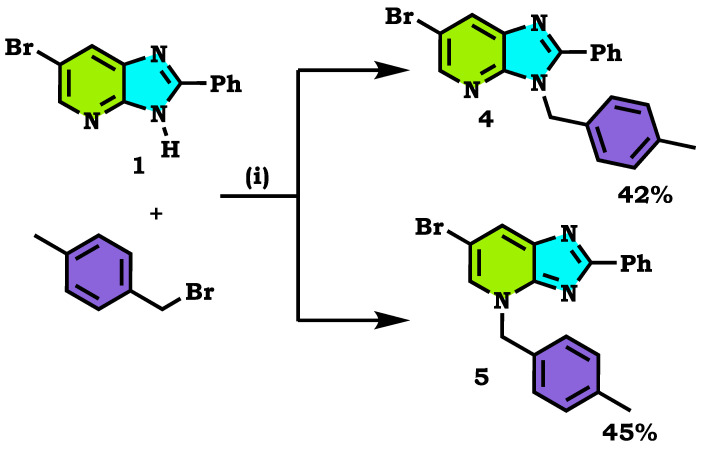
Synthetic route of novel imidazo[4,5-b]pyridine derivatives and conditions (**4**, **5**): (i) DMF (15 mL), K_2_CO_3_ (1.2 equiv), t-BAB (0.1 equiv), 6 h, RT.

**Scheme 4:**
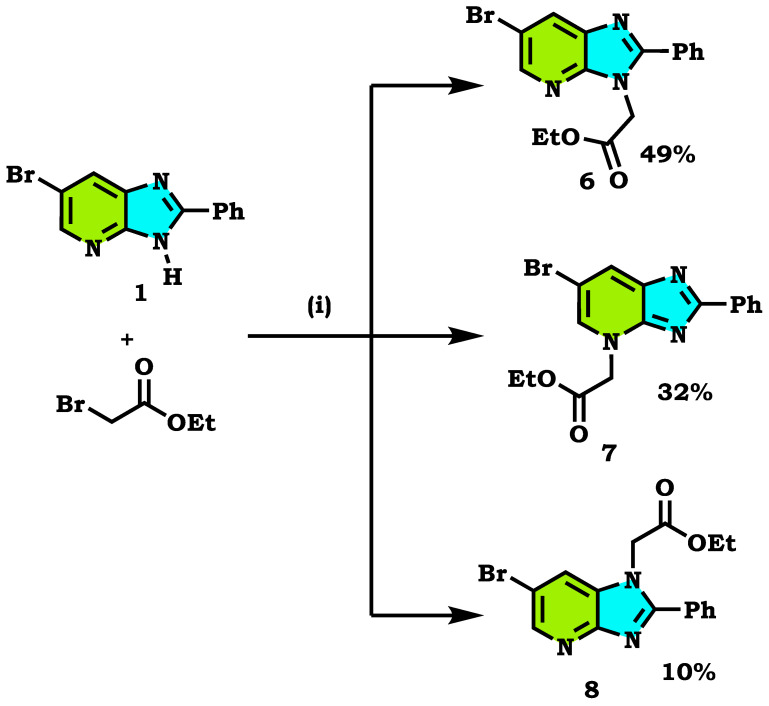
Synthetic route of novels imidazo[4,5-b]pyridine derivatives (**6**–**8**) and conditions: (i) DMF (15 mL), K_2_CO_3_ (1.2 equiv), t-BAB (0.1 equiv), 6 h, RT.

The alkylation reactions of imidazo[4,5-b]pyridine are very important pathways in the synthesis of some new imidazo[4,5-b]pyridine derivatives. In a continuation of our ongoing work devoted to the preparation and application of new imidazo[4,5-b]pyridine derivatives [28,29,30,31], we report here the synthesis of new imidazo[4,5-b]pyridine derivatives by the action of (1-(chloromethyl) benzene, 1-(bromomethyl)-4-methylbenzene and ethyl 2-bromoacetate on the 6-bromo-2-phenyl-3H imidazo[4,5-b]pyridine under phase transfer catalysis conditions. The reaction led to effects on two positions: the nitrogen at position 3 (N^3^) and at position 4 (N^4^). On the other hand, the action of ethyl 2-bromoacetate affected the nitrogen atom in the first position (Scheme 1, Scheme 2, Scheme 3 and Scheme 4).

We continued our experiments to apply this method for the preparation and the study of the antimicrobial activity of imidazo[4,5-b]pyridine derivatives.

The tautomeric form present in the imidazo[4,5-b]pyridine skeletons (1) made this system more diversified, and the condensation of 6-Bromo-2-phenyl-3H-imidazo[4,5-b]pyridine with 1.2 equivalents of alkyl halides under (PTC) conditions led to effects on two positions: the nitrogen at position 3 (N^3^) and at position 4 (N^4^), (Scheme 2 and Scheme 3). ON the other hand, the action of ethyl 2-bromoacetate affected the nitrogen atom in the first position (Scheme 4). All the obtained compounds (2–8) were purified by column chromatography and isolated with good overall yields (10–49%). The molecular structures of the new compounds were established on the basis of the NMR spectroscopic data (Figures S1–S6; Supplementary Materials), mass spectrometry and XRD single crystal for **2** (Scheme 2).

#### 2.1.1. Crystallographic Data

**Figure 2:**
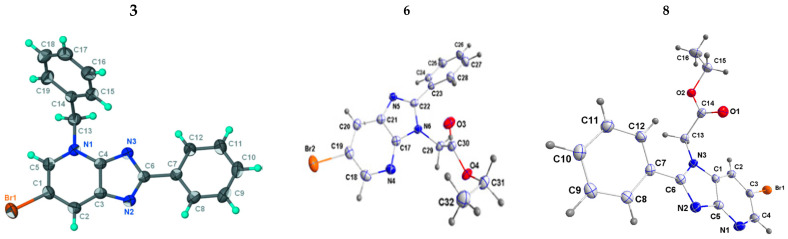
View ORTEP of compounds **3**, **6** and **8** [32].

Studies on the crystallographic data of compound (**3**) show that alkylation took place at the third position [32]. Additionally, the crystallized form for **3** and **6** is a monoclinic system (Figure 1, Tables S1–S3; Supplementary Materials). Moreover, the H-bond interactions for compounds **3**, **6** and **8** are listed (Figure 1, Table S4; Supplementary Materials). Furthermore, the crystallographic study for compound (**3**) confirms well that alkylation took place at the fourth position (Figure 1). Therefore, the synthesis of the imidazopyridine skeletons was confirmed [33] by a crystallographic study performed for compounds **3** and **6** (Figure 2):

#### 2.1.2. The Hirschfield Profile for Molecular Packing

**Figure 3:**
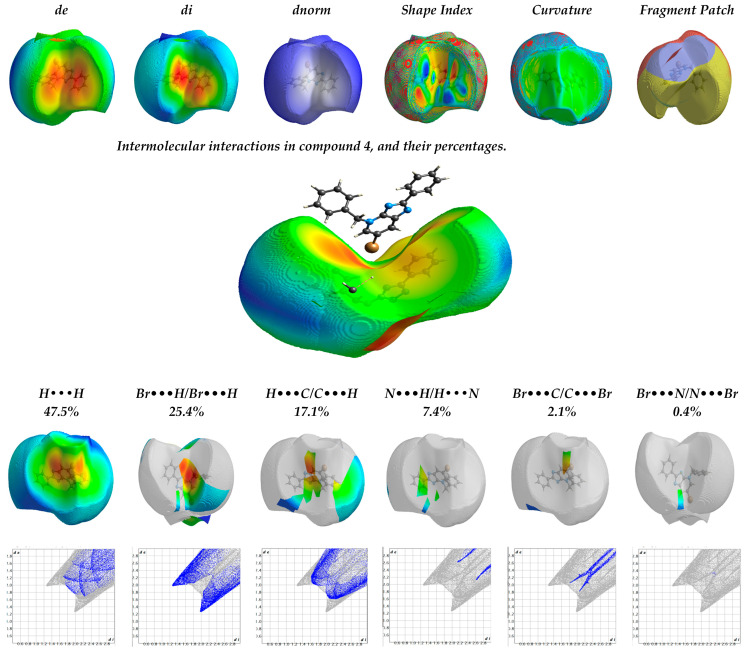
HF and 2D interaction diagrams and their impact in compound **3**.

**Figure 4:**
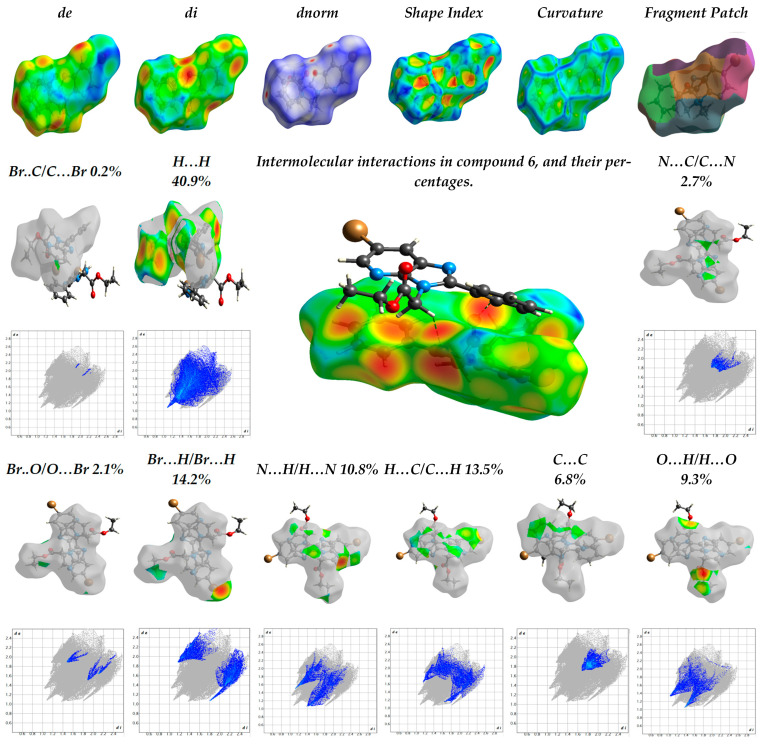
The HFM 2D interaction diagrams and their contribution to compound **6,** as represented in the d_norm_, d_i_, d_e_, shape index “si”, curvedness “crv” and fragment batch “fb”.

**Figure 5:**
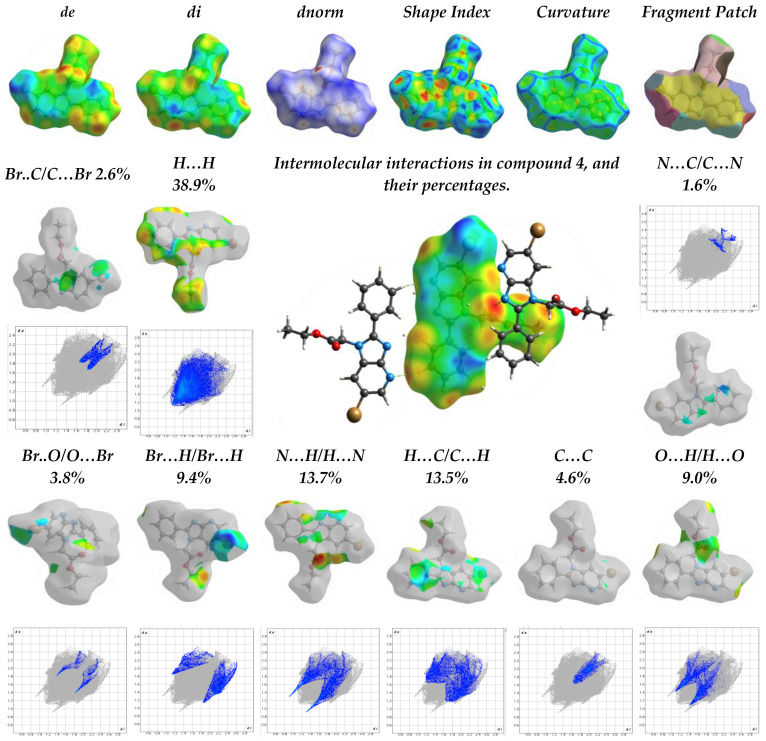
The HFM 2D interaction diagrams and their contribution in compound **8,** as represented in the *d_norm_*, *d_i_*, *d_e_*, *shape index “si”*, *curvedness “crv”* and *fragment batch “fb”*.

All intermolecular patterns and Hirschfield-surfaces “HF” that shared in the stabilization of **3**, **6** and **8** molecular packing were mapped in Figure 3, Figure 4 and Figure 5. The dnorm was calculated as (dnorm = (di − rvdWi)/rvdWi + (de − rvdWe)/rvdWe) [34]. The rvdWi and rvdWe were related to the tightest interaction between internal and exterior particles morphology through VanderWaals radii. The “+” dnorm accepts short rvdW, but lengthy rvdW have “−” value. The correlation between the “de and di” of the HF for **3**, **6** and **8** was obtained using the crystal explorer [35].

3D–HF for molecule **3** is mapped in Figure 3, over (0.1866 Å to 8.1945 Å) for dnorm, (1.6.17 Å to 6.6658 Å) for di, (1.2618 Å to 11.0466 Å) for de, (−1.00 Å to 1.00 Å) for shape-index, (−4.00 Å to 4.00 Å) for curvedness and (0.00 Å −3.00 Å) for patch fragment, respectively. The red area in the dnorm fingerprint represents the H-interactions, which extend beyond the vdWs-radii. Molecule **3** was packed by the shortest interactions for Br•••N/N•••Br (0.4%) and Br•••C/C•••Br (2.7%), while the highest interactions at Br•••H/H•••Br (25.4%) and C•••H/H•••C (17.1%) contacts contributed in the crystal packing. H•••H are the most prevalent, with 47.5% of the region covered by the maps.

3D–HF for molecule **6** is mapped in Figure 4, including (0.166 Å to 1.4059 Å) for dnorm, (0.9196 Å to 2.637 Å) for di, (0.9176 Å to 2.5592 Å) for de, (−1.00 Å to 1.00 Å) for shape-index, (−4.00 Å to 4.00 Å) for curvedness and (0.00 Å −15.00 Å) for patch fragment, respectively. The shortest interactions were for Br•••C/C•••Br (0.6%) and N•••C/C•••N (2.7%). The Hydrogen contacts signified by a red area for the dnorm fingerprint were more intense than the radii of vdWs, as H…C/C…H (13.5%), O•••H/H•••O (9.3%) and N•••H/H•••N (10.8%) contacts contributed to the **6** molecule for the crystal packing. H•••H contacts were the most dominant, with 40 % from the whole maps area, while π•••π interaction contributed to crystal packing by (6.8%).

Compound **8** in (Figure 5) included (0.166 Å to 1.4059 Å) for *dnorm*, *di* (0.9196 Å to 2.637 Å) for *di*, (0.9176 Å to 2.5592 Å) for *de*, (−1.00 Å to 1.00 Å) for shape-index, (−4.00 Å to 4.00 Å) for curvedness and (0.00 Å −15.00 Å) for patch fragment, respectively. The shortest interactions were for Br•••C/C•••Br (2.6%) and N•••C/C•••N (1.6%). The Hydrogen contacts as red zone, which were represented in dnorm fingerprint, had interactions of higher intensity than the vdWs radii, as **H…C/C…H (13.5%),** O•••H/ H•••O (9.3%) and N•••H/H•••N (10.8%) contacts contributed to the crystal packing. The H•••H were the most dominant, with 40.9% from the whole maps area, while the π•••π interaction contributed to crystal packing by (6.8%).

Si is a sensitive indicator of any lattice shape deviation (Figure 3, Figure 4 and Figure 5). Red triangles are used to symbolize the concave region, which was located on the particle’s upper plane, indicating 6-bromo-2-phenyl-3H-imidazol outside of the surface for **3**, **6** and **8**. The triangles with blue highlights show the location of the phenyl fragment on the superficial exterior. SI data were in agreement with the 2D pattern. The morphology for the particles’ surface for **3**, **6** and **8** was studied, together with the crv fingerprint, which separated into dual patches of curvature due to the connections between nearby molecules.

### 2.2. Molecular Modelling Study

#### 2.2.1. Tautomerization Structure and Optimization Geometry

**Figure 6:**
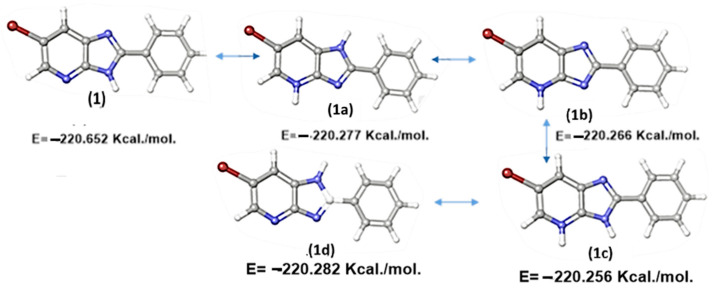
Optimized structures of the tautomeric structures (**1**–**1d**)**.**

Imidazo[4,5-b]pyridine for **1a**–**1d** tautomerism is an important key for chemical and biochemical investigations. Tautomerism, isomerism, and opening–closing heterocyclic rings are present in many medications, and they play a significant part in drug development for biomolecules with biological activity [36]. All potential tautomer-structures for **1** molecule were optimized by DFT/ B3LYP/6311G** (Figure 6). The **1** canonical structure with (−220.652 kcal/mole) was considered to have the lowest minimization energy with the utmost stable tautomer form. The calculated total energy values are corrected by obtained zero-point energy. One can arrange the order of stability as **1c** < **1b** < **1a** < **1d** < **1**. The H atom, which attached to N of imidazole in position three, is preferable to one in postion 4 and N of pyridine. The bonding of the movable H to pyridine (**1**) was most stable form (Figure 6). The presence of the pyridine group closer to imidazole led to a decreased lengthening bond between imidazole and the phenyl scaffold, and hence a stronger holding of the phenyl ring and more ability for interaction during the chemical reaction. The dihedral angles were asymmetrical for both NH, owing to the electrostatic repulsive interactions with the neighboring hydrogens. Thus, we could manage the alkylation reactions of imidazopyridine (**1**).

**Figure 7:**
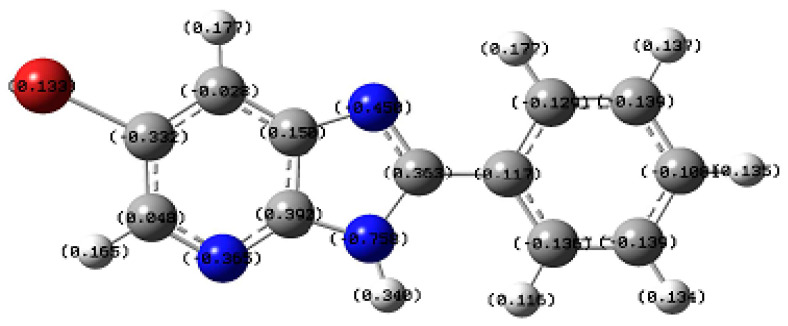
Optimized structure of compound (**1**).

Figure 7 showed the electron density of the compound (**1**), which illustrated that the negative charge localized on the nitrogen atom at the third position was greater than that at the fourth position, while the negative charge shown on nitrogen at the first position was the lowest. These results explain the difference in reactivity of the various nitrogen atoms of compound (**1**) towards the carbocations.

The optimizations of the energies obtained for each pair of regioisomers (third and fourth positions) are grouped in Table 1. From an energy standpoint, in Table 2, we found that imidazopyridines alkylated at the third position were more stable than their analogues alkylated at the fourth position when the alkylating agent was not bulky or so far away. In fact, (**3**) and (**5**) alkylated by the benzyl derivatives at the fourth position were more stable, respectively, than (**2**) and (**4**) alkylated in the third position, which explains that the volume of carbocation directs the alkylation towards the pyridine nitrogen because of the steric gene created with phenyl in the second position. Practically, the yields of regioisomers (**2**) and (**4**) alkylated at the third position were almost equal to yields of regioisomers alkylated in the fourth position. Therefore, theoretical results are in good agreement with the experimental findings.

#### 2.2.2. Analysis of Frontier Molecular Orbitals’ FMOs and Electronic Reactivity Descriptors

**Figure 8:**
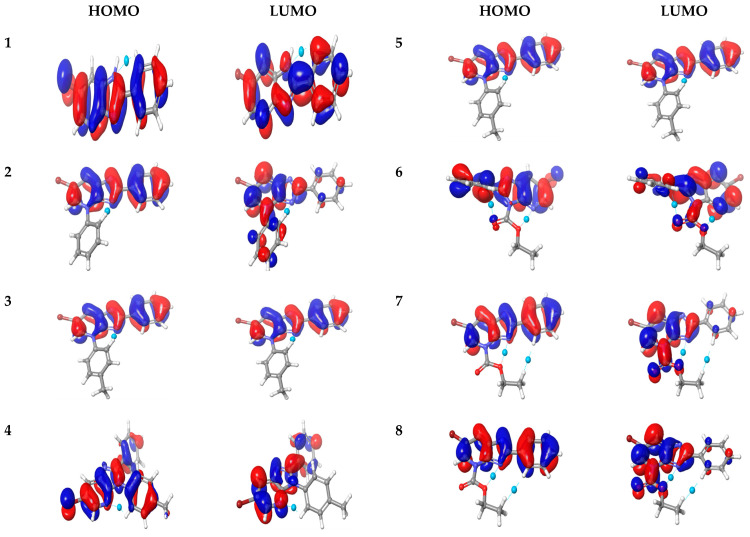
HOMO and LUMO orbitals for phenyl-3H-imidazo[4,5-b]pyridine derivatives that were simulated by TD-DFT/B_3_LYP/6-311G** using 0.032.

The DFT/B3LYP/6311G** was applied to compute the energy gap “**Δε**” for **1**–**8** molecules using distribution shapes along the following orbitals: HOMO_“*donating electrons*”_ and LUMO_“*accepts electrons*”_ (Figure 4). The **Δε** was able to determine a molecule’s kinetic stability before simulating its chemical reactivity [37]. Imidazo[4,5-b]pyridine with a high **Δε** has a hardness quality “η” and is a good nucleophile; the pyridine system with a low **Δε** is a soft hybrid and an excellent electrophile ω (Table 2). In addition, the ionization potential (IP), electronegativity “χ” and global electrophilicity “ω” were estimated as additional electronic characteristics related to energy gap, and are shown in Table 2. The calculated **Δε** = 0. 105 to 0.177 au. for 6-bromo-2-phenyl-3H-imidazo[4,5-b]pyridine hybrids **1**–**8** and the reported biomaterials values were harmonized [38]. HOMO was distributed in **1**–**8** over the phenyl-3H-imidazo[4,5-b]pyridine fragment. These orbitals transferring into the LUMO orbital over the imidazole center (Figure 8). The HOMO→LUMO orbital transfer in **1**–**8** took place over the phenyl-3H-imidazo[4,5-b]pyridine Skelton. In compound **8**, the HOMO and LUMO orbitals shielded between phenyl-3H-imidazo[4,5-b]pyridine and the pyridine fragment.

In addition, ω had a low value of between 0.292 and 0.616 au., which directed the powerful stabilization efficiency which was produced from the electrons in outer space. Compounds 2, 4 and 6 had more stabilization than 3, 5, 7 and 8. The qualifications value for η exhibited a low aptitude transformation of electronic current to distortion direction. As expected [39], the anticancer efficiency for biomolecules renationalized directly with the antioxidant power, which was related to the small value of the ionization potential (IP) [38]. The molecule had scavenging ability due to the transfer one electron mechanism, and a healthier antioxidant [39]. The antioxidant power rose as the IP value dropped. The tested compounds showed a low IP = 0.139 to 0.237 au.

#### 2.2.3. Molecular Electrostatic Potential “MEP” Fingerprint

**Figure 9:**
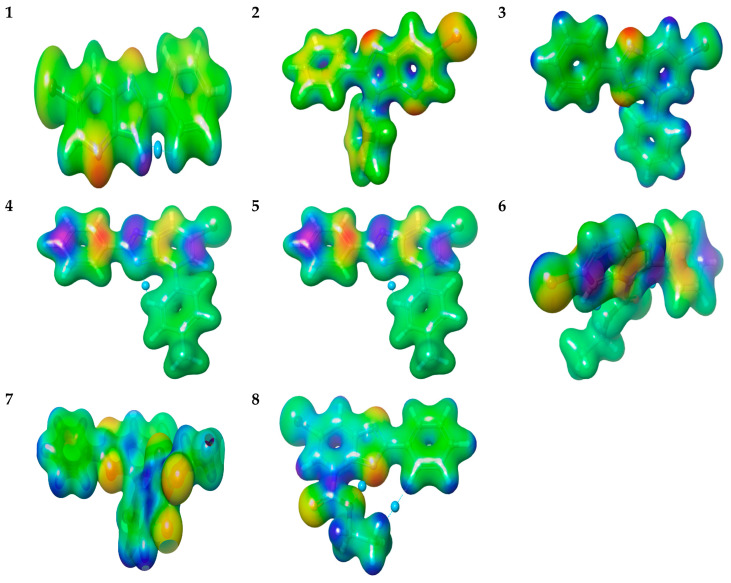
Molecular electrostatic potential map (0.032 contour value) and intramolecular noncovalent interactions for (**1**–**8**).

MEP is a signature for the polarization of the outer electrons and the distribution of those electrons in relation to the molecular environment’s reactivity and ability to interact with H-atoms. Additionally, it provides complete information on electrophilic and nucleophilic chemical locations. As a result, we can graphically determine the statistical polarity through variations in color; see Figure 9, which served to distinguish the polar (“−” charge as red color) and nonpolar (“+” charge as blue color) molecular zones. The green zone was noted as having a potential that was halfway between the dual red and blue. The order of red, yellow, blue and green rose as electrostatic potential levels changed in the colors’ distribution on MEP (Figure 9). The electron distribution supported the idea that the compounds **1**–**8** were able to attack the DHFR bacterial enzyme based on size and shape. Figure 9 showed that the yellow region condensed over the imidazole ring in all hybrids, which caused an increase in the electrophilicity effect. The blue highlight extended over the substant of imidazole for all compounds, which activated the nucleophilicity of the pyridine cores**,** which handle the substrate’s capacity to identify the binding site via electrostatic interaction with the receptor.

### 2.3. Molecular Docking Profile

To verify the relationship between the biological findings in vitro and the interaction affinities of the investigated hybrids, the docking analysis of the most active derivatives, **2**, **4** and **6**, was assessed to identify their binding mode inside the (PDB: 1DLS) [40] and DNA gyrase (PDB; 4uro [41,42,43]) active sites, as well as the structural orientation and conformation. The docking steps were applied as in a previously reported method [44,45]. The 3D loop of dihydrofolate-reductase “DHFR” was created using the mGenTHERADER, which utilized the docking framework. Herein, we established the toxicity behavior in binding energy BE terms for the tested compounds over DHFR and DNA gyrase receptors, then compared with reference inhibitors (Methotrexate and Novobiocin). The investigated compounds were re-docked, and achieved a root mean square deviation (RMSD) of less than 2 Å.

The Methotrexate targeted vital amino acids (GLU30, ILE7, VAL115, LYS 68, ARG70, LYS68, ARG70, ILE7 and PHE34) in the DHFR binding pocket. Novobiocin interacted with important amino acids residues (ASN54, GLU58, Pro87, ASP81, ASP89 and ARG144) in the DNA gyrase active site.

The binding efficiency “ΔE” was evaluated using the fingerprint interaction between ligand and protein (PLIF). Table 3 shows all of the docking experiment’s energy values. The poses were generated by the “Oples3e” molecular mechanics force-field. In order to assess the binding affinities of **1**–**8** molecules, the pose which had the lowest “ΔE and RMSD” was chosen. To further validate the “ΔE”, the inhibitory constant “Ki” and ligand-efficiency “LE” were computed [46].

Structurally, the tested derivatives possessed the imidazo[4,5-b]pyridine backbone. The chief difference between the chemical structures lay in the alkylation site, which could play a crucial role in its biological activities. The ΔE variation with regard to DHFR and DNA gyrase, as seen in the present investigation, may be caused by structural variations.

**Figure 10:**
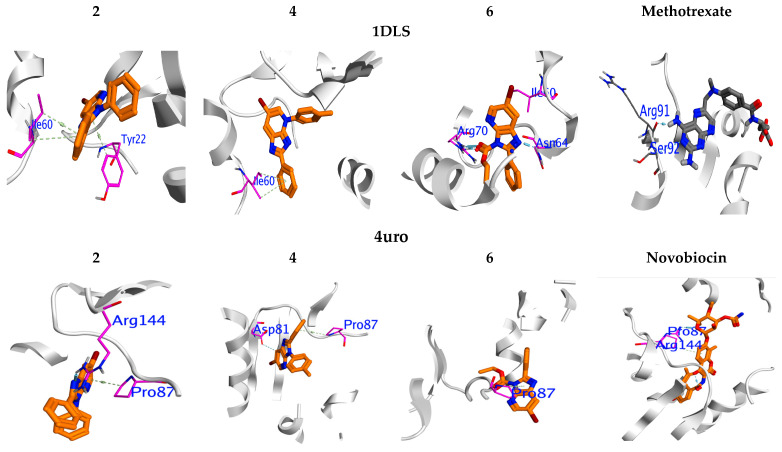
Three-dimensional mapping surface for **2**, **4**, **6** and reference inhibitors inside the active sites of PDB:1DLS and PDB: 4uro.

The Methotrexate “original inhibitor” against DHFR displayed a binding energy of ΔE =  −7.85 kcal/mol with Ki  =  1.88 A, through two H-bonds sidechains with Arg91 and Ser92. The binding efficiency was arranged for most active compounds as **4** > **6** > **1**, with a promising inhibition constant ranging between 2.16 and 1.98. Molecule **2** showed ΔE = −7.00 kcal/mol, and was stabilized in the binding site by the arrangement of the iIle 60 with the imidazole ring with perpendicular mode through the formation of a sticky π–π bond. 6-bromo-2-phenyl-3-(p-tolyl)-3H-imidazo[4,5-b]pyridine (**2**) formed an extra π–π bond with Tyr22, while ethyl 6-bromo-2-phenyl-3H-imidazo[4,5-b]pyridine-3-carboxylate (**6**) formed two strong H-bonds with Asn64 and Arg70 (Figure 10). In addition, all the bioactivity metrics LE and Ki were within a normal range for **1**–**8** [47]. It can be inferred that the molecular docking encourages us to perform antimicrobial activity against most binding efficiencies in the docking experiment.

The original inhibitor Novobiocin displayed a binding energy of ΔE = −7.24 kcal/mol with Ki = 1.88 A against DNA gyrase, through an H-bonds sidechain with Pro87 and a π–π interaction with Arg144. The binding efficiency was arranged for most active compounds as **6** > **4** > **1**, with a promising inhibition constant ranging between 2.19 and 1.93. Molecule **2** showed ΔE = −6.27 kcal/mol, and was stabilized in binding site by the arrangement of the Pro87 with the imidazole ring in perpendicular mode through the formation of a sticky π–π bond. 6-bromo-2-phenyl-3-(p-tolyl)-3H-imidazo[4,5-b]pyridine (**2**) formed an extra H-bond with Arg144, while compound **6** formed a π–π bond with Pro87 (Figure 10). In addition, all the bioactivity metrics of LE and Ki were within a normal range for **1**–**8** [47]. It can be inferred that the molecular docking encouraged us to perform antimicrobial activity against most bending efficiencies in the docking experiment.

### 2.4. Biological Activity

Many pharmaceutical compounds have two or more space isomers. The pharmacological activity of racemic pharmaceutical preparations is usually associated with the effect of only one regioisomer [48]. In this work, we chose to perform the antibacterial test on imidazo[4,5-b]pyridine derivatives N^3^-alkylated from each reaction (**4** and **6**) towards two different classes of bacterial strains (Gram-positive bacteria (***Bacillus cereus***) and Gram-negative bacteria (***Escherichia coli***)).The table below shows the activity of tested products 4 and 6 (Table 4). The detection of the antibacterial capacity of synthesized products was carried out by the disk diffusion method. The results obtained from this activity are illustrated in the form of the presence or absence of inhibition zones (Table 4).

For the tested products, *B. cereus* was more sensitive to the action of all products compared to *E. coli*, which was much more resistant, with a total growth in the presence of product (**4**). Only product (**6**) was able to inhibit the growth of *E. coli.* The antibacterial activity against the studied strains was evaluated by observing their inhibitions in direct contact with the products tested at different concentrations, using the microdilution method. The MIC values are set out in Table 4.

As can be seen from Table 5, compound **2** had antimicrobial power to varying degrees depending on the microbial strain tested. Among the two tested strains, it is interesting to note that *B. cereus* was the most sensitive against all the studied compounds, in particular (**2**), which exerted the strongest inhibitory effect with a minimum inhibitory concentration of 0.07 Mg/mL and 0.315 mg/mL, respectively, which confirms the results obtained by the agar diffusion method.

## 3. Materials and Methods

### 3.1. Chemistry

The characterization of the prepared imidazopyridine derivatives by ^1^H NMR (300 MHz) and ^13^C NMR (75 MHz) spectra were recorded on Bruker spectrometers using CDCl_3_ and DMSO-*d_6_* as solvents. The coupling constants (J) were expressed in Hertz (Hz). Multiplicities are reported as follows: singlet (s), doublet (d), doublet of doublets (dd), triplet (t) and multiplet (m). Melting points (mp) were recorded on a Kofler bench, and were not corrected. Flash chromatography was conducted using flash silica gel 60 (Merck 230–400 mesch). TLC (thin layer chromatography) was used to monitor the reaction progress.

Synthesis of 6-Bromo-2-phenyl-3H-imidazo[4,5-b]pyridine (**1**):

To 5.31 mmol of 5-bromo-2,3-diaminopyridine (1 g) dissolved in 40 mL of ethanol (EtOH), 5.84 mmol of benzaldehyde (0.6 mL) was added dropwise, and 0.531 mmol of diiodide (0.09 g). The solution was brought to reflux with magnetic stirring (90 °C). Over 24 h, a brown solid was formed, filtered and washed 3 times with distilled water and then dried in an oven.

Brown solid, m.p > 260 °C, Rf: 0.48 (eluent: ethyl acetate/hexane (1/2)), Yield: 80%, ^1^H NMR (DMSO-d_6_) δppm: 8.42–7.57 (m, 7H, (2H_pyr_ + 5H_arom_)); 13.76 (s, 1H, N-H). ^13^C NMR (DMSO) δppm: 113.43 (Cq); 127.39, 127.55, 129.54, 131.40, (CHAr); 129.22, 129.54, 129.66 (Cq).


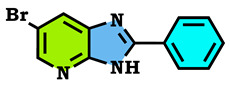


General alkylation procedures of 6-Bromo-2-phenyl-3H-imidazo[4,5-b]pyridine:

Then, 0.9 mmol of 6-bromo-2-phenylimidazo[4,5-b]pyridine (0.25 g), 20 mL of DMF and 1.35 mmol of K_2_CO_3_ (0.186 g) were placed in a two-necked round bottom flask equipped with a magnetic stirrer with stirring for 5 min; next, 0.18 mmol of tetra-N-butylammonium bromide (t-BAB) (0.058 g) was added, and then 1.08 mmol of appropriate mono-halogenated compounds including (1-(chloromethyl) benzene, 1-(bromomethyl)-4-methylbenzene and ethyl 2-bromoacetate. The reaction was brought to room temperature for 6 h. After the removal of salts by filtration, DMF was evaporated under pressure and the residue obtained was dissolved in dichloromethane. The rest of the salts were removed by washing the organic phase three times with distilled water, and the traces of water in the organic phase were eliminated by the desiccant Na_2_SO_4_. After filtration, the dichloromethane was evaporated (not to dryness) and the product obtained was separated by chromatography on a column of silica gel (eluent: ethyl acetate/Hexane (1/2)). Our alkylated products were isolated.

3-Benzyl-6-bromo-2-phenyl-3H-imidazo[4,5-b]pyridine (**2**)

Yellow solid, m.p = 74 °C, Rf: 0.64 (eluent: ethyl acetate/hexane (1/2)), Yield: 43%, ^1^H NMR (CDCl_3_) δppm: 5.64 (s, 2H, CH_2_); 6.97–6.99 (m, 2H, H_Ar_); 7.52–7.57 (m, 3H, H_Ar_); 7.22–7.28 (m, 3H, H_Ar_); 7.74–7.77 (m, 2H, H_Ar_); 8.46 (d, 1H, H_Ar_, J = 1.8 Hz); 8.49 (d, 1H, H_Ar_, J = 2.1 Hz); ^13^C NMR (CDCl_3_) δppm: 46.83 (C, CH_2_); 114.09 (C_q_); 126.67, 127.98, 129.20, 129.35, 129.39, 129.76, 129.80, 131.10 (CH_Ar_); 136.31, 137.07 (C_q_); 144.68 (CH_Ar_); 147.88, 156.00 (C_q_).


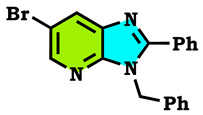


4-Benzyl-6-bromo-2-phenyl-4H-imidazo[4,5-b]pyridine (**3**)

Yellow crystal, m.p = 178 °C, Rf: 0.27 (eluent: ethyl acetate/hexane (1/2)), Yield: 44%, ^1^H NMR (CDCl_3_) δppm: 5.88 (s, 2H, CH_2_); 7.32–7.40 (m, 3H, H_Ar_); 7.48–7.51 (m, 3H, H_Arom_); 7.58–7.61 (m, 2H, H_Arom_); 8.38–8.43 (m, 3H, H_Arom_); 8.70 (d, 1H, H_Arom_, J = 1.6 Hz). ^13^C NMR (CDCl_3_) δppm: 56.70 (CH_2_); 105.88 (Cq); 128.31, 129.00, 129.03, 129.07, 129.27, 130.31, 130.59, 131.51, (CH_Arom_); 134.53, 135.90, 146.40, 153.60 (C_q_).


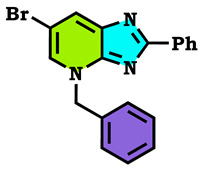


6-Bromo-3-(4-methyl-benzyl)-2-phenyl-3H-imidazo[4,5-b]pyridine (**4**)

Yellow solid, m.p = 142 °C, Rf: 0.76 (eluent: ethyl acetate/hexane (1/2)), Yield: 42%, ^1^H NMR (CDCl_3_) δppm: 2.32 (s, 3H, CH_3_); 5.55 (s, 2H, N-CH_2_); 6.96–7.11 (dd, 4H); 7.44–7.71 (m, 5H, H_Arom_); 8.24 (d, 1H, H_pyr_); 8.46 (d, 1H, H_pyr_). ^13^C NMR (CDCl_3_) δppm: 21.03 (CH_3_); 46.81 (N-CH_2_); 114.27 (Cq); 135.22, 133.39 (Cq); 126.51, 128.83, 129.26, 129.53, 129.59, (CH_Arom_); 144.89, 130.56 (CH_Arom_); 147.13, 137.55 (Cq); 155.69 (Cq).


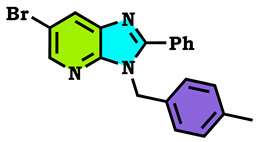


6-Bromo-4-(4-methyl-benzyl)-2-phenyl-3H-imidazo[4,5-b]pyridine (**5**)

White solid, m.p = 160 °C, Rf: 0.33 (eluent: ethyl acetate/hexane (1/2)), Yield: 45%, ^1^H NMR (CDCl_3_) δppm: 2.38 (s, 3H, CH_3_); 5.83 (s, 2H, N-CH_2_); 7.23–7.39 (dd, 4H_Arom_, J = 2.4 Hz); 8.20–7.46 (m, 5H, H_Arom_); 8.52 (d, 1H, H_pyr_); 8.64 (d, 1H, H_pyr_). ^13^C NMR (CDCl_3_) δppm: 21.24 (CH_3_); 56.56 (N-CH_2_); 106.09 (Cq); 130.82, 130.41, 128.43 (CH_Arom_) 133.97, 139.42, 146.77, 154.13, 154.78 (Cq).


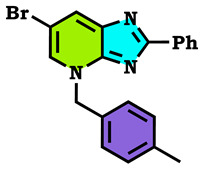


Ethyl 2-(6-bromo-2-phenyl-3H-imidazo[4,5-b]pyridin-3-yl)acetate (**6**):

Colorless crystals, m.p = 120 °C, Rf: 0.6 (eluent: ethyl acetate/hexane (1/2)), Yield: 49%, ^1^H NMR (CDCl_3_) δppm: 1.22 (t, 3H, CH_3_); 4.23 (m, 2H, (CH_2_-O)); 5.07 (s, 2H, N-CH_2_); 7.74–7.53 (m, H, 5H_Arom_); 8.22 (d, 1H, H_pyr_); 8.43 (d, 1H, H_pyr_). ^13^C NMR (CDCl_3_) δppm: 14.02 (CH_3_); 44.84 (CH_2_-O); 62.14 (N-CH_2_); 114.52 (Cq); 129.05, 129.82 (5CH_Arom_); 130.75, 144.84 (2CH); 136.18, 147.33 (Cq); 155.96 (C-Br); 167.46 (C=O).


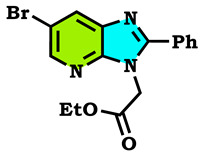


Ethyl 2-(6-bromo-2-phenyl-1H-imidazo[4,5-b]pyridin-4-yl)acetate (**7**)

Reddish solid, m.p = 126 °C, Rf: 0.44 (eluent: ethyl acetate/hexane (1/1)), Yield: 32%, ^1^H NMR (CDCl_3_) δppm: 0.80 (t, 3H, CH_3_); 4.19 (q, 2H, (CH_2_-O)); 5.46 (s, 2H, N-CH_2_); 7.36–8.01 (m, 5H, H_Arom_); 8.13 (d, 1H, H_pyr_); 8.45 (d, 1H, H_pyr_). ^13^C NMR (CDCl_3_) δppm: 14.01 (CH_3_); 38.61 (CH_2_-O); 50.43 (N-CH_2_); 114.34 (Cq); 130.51, 119.78 (CH_Arom_); 142.84 (CH_Arom_); 165.26 (C=O).


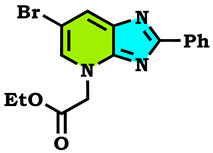


Ethyl 2-(6-bromo-2-phenyl-1H-imidazo[4,5-b]pyridin-1-yl)acetate (**8**)

Reddish crystals, m.p = 140 °C, Rf: 0.35 (eluent: ethyl acetate/hexane (1/1)), Yield: 10%, ^1^H NMR (CDCl_3_) δppm: 0.89 (t, 3H, CH_3_); 4.32 (q, 2H, (CH_2_-O)); 5.59 (s, 2H, N-CH_2_); 8.07–7.57 (m, 5H, H_Arom_); 8.22 (d, 1H, H_pyr_); 8.47 (d, 1H, H_pyr_). ^13^C NMR (CDCl_3_) δppm: 14.03 (CH_3_); 39.40 (CH_2_-O); 50.52 (N-CH_2_); 144.79 (CH_Arom_); 114.54 (Cq); 131.21, 127.48 (CH_Arom_); 167.46 (C=O).


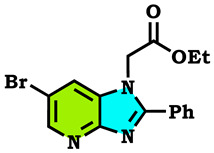


### 3.2. Theoretical Study

The theoretical calculation was performed for the energies of each tautomeric form and the electron density of each nitrogen. The optimization results of the energies obtained for each pair of regioisomers (3rd and 4th position) were obtained with the Density functional theory method (DFT) using the level B3LYP [49] with 6311G** basis set. Gaussian 03 was used for optimizing the geometry for the structures by standard methods [50].

Docking study:

Molecular docking for target compounds into DHPS using GOLD (version 5.2) was achieved. H_2_O and the original inhibitor were removed from the obtained DHFR crystal structure and DNA gyrase, then H atoms were added. The **2**–**8** ligands were redocked against the vacant active site. The charges were allocated using the Charm force field, and the ChemPLP scoring function was created for measuring the binding affinity.

### 3.3. Biology

Antibacterial activity:

The antibacterial activity of imidazo[4,5-b]pyridine derivatives 4 and 6 was evaluated according to the disk-diffusion method [51] against one representative of each class of susceptible strains: Gram negative strain (*Escherichia coli*) and Gram positive strain (*Bacillus cereus*) using Mueller Hinton agar (MHA) medium. Plates were pre-incubated at 37 °C for 24 h. Then, 100 µL of microbial inoculum adjusted to 0.5 McFarland was spread on the plate’s surfaces using a sterile glass rod to prepare microbial lawns. A sterile paper disk (6 mm in diameter) was placed on the surface of each agar plate, and impregnated with 10 µL of each imidazo[4,5-b]pyridine solution (4 and 6) at a final concentration of 100 µg/disk. Then, Petri dishes were incubated at 37 °C for 24 h. The diameters of the inhibition zones were measured in mm (including disk diameter) with calipers. A disk impregnated with dimethylsulfoxide at 2% was used as a negative control. Each experiment was carried out in triplicate.

Minimum inhibitory concentration determination (MIC) against bacterial strains

The MIC was performed in a 96 well-microplate using the microdilution assay according to the protocol previously described by Chraibi et al. [52], with slight modifications. Briefly, a stock solution of each product was prepared in (DMSO). Then, serial dilutions of all tested products were prepared in Mueller Hinton Broth medium (MHB) at final concentrations ranging between 5 mg/mL and 0.0025 mg/mL. The 12th well was considered as growth control (free drug control). Afterwards, 50 µL of bacterial inoculum was added to each well at a final concentration of 10^6^ CFU/mL. After incubation at 37 °C for 24 h, 10 µL of rezasurin was added to each well as a bacterial growth indicator. After further incubation at 37 °C for 2 h, the bacterial growth was revealed by the change of coloration from purple to pink. Experiments were carried out in triplicates.

## 4. Conclusions

The synthesis of a series of imidazo[4,5-b]pyridine derivatives was realized with good yields, using alkylation under conditions of phase transfer catalysis solid–liquid (CTP).The structures of the obtained compounds were confirmed by NMR spectroscopy (1H and 13C) and X-ray diffraction. HF analysis unveiled the interaction types’ stabilized crystalline phase for compounds **3**, **6** and **8**, including Br•••C/C•••Br, N•••C/C•••N, H…C/C…H, O•••H/H•••O and N•••H/H•••N interactions. The theoretical results produced by the DFT method were in good agreement with the experimental results. The MESPs demonstrated that pyridines are nucleophiles, while imidazoles exhibit an electrophilic character. To determine the potential mode of interactions with the DHFR active site, the three N1, N3 and N4 regioisomers were further subjected to a molecular docking study. The outcomes demonstrated that these active analogues engaged in a number of significant interactions with the target enzyme’s active regions. The antimicrobial activity of the tested compounds was qualitatively and quantitatively assessed by the disk-diffusion and microdilution methods, which showed that *B. cereus* was the most sensitive against all the studied compounds, while *E. coli* was the most resistant strain. Thus, the present study demonstrated the synthesized products to be potential antimicrobial agents with further modification.

## Figures and Tables

**Table 1:** Total energies of all synthesis imidazo[4,5-b]pyridine derivatives (**2**–**8**).

*Compound Alkylated at Third Position*	*Energy E3* (Kcal/mol.)	*Compound Alkylated at Fourth Position*	*Energy E4* (Kcal/mol.)	*Compound Alkylated at First Position*	*Energy E1* (Kcal/mol.)
** 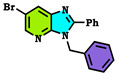 **	−298.122 Kcal/mol.	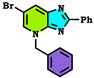	−263.047 Kcal/mol.	T	ND
** 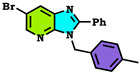 **	−294.781 Kcal/mol.	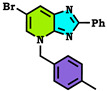	−260.099 Kcal/mol.	T	ND
** 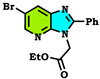 **	−297.763 Kcal/mol.	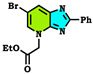	−262.738 Kcal/mol.	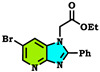	−263.756 Kcal/mol.

T: trace; ND: Not detected.

**Table 2:** Energetic chemical descriptors for **1**–**8** derivatives.

	_HOMO_	_LUMO_	Δε	η	S	*IP*	μ	χ	ω	Δ*N_max_*
**1**	−0.225	−0.053	0.173	0.086	11.563	0.225	−0.095	−0.139	0.292	−0.803
**2**	−0.216	−0.082	0.135	0.067	14.819	0.139	−0.115	−0.149	0.403	−1.104
**3**	−0.224	−0.073	0.151	0.075	12.806	0.159	−0.110	−0.148	0.345	−0.935
**4**	−0.228	−0.054	0.174	0.087	11.480	0.228	−0.097	−0.141	0.299	−0.810
**5**	−0.237	−0.060	0.177	0.089	11.271	0.237	−0.104	−0.148	0.322	−0.836
**6**	−0.220	−0.115	0.105	0.053	18.993	0.148	−0.141	−0.167	0.616	−1.590
**7**	−0.223	−0.101	0.122	0.061	16.442	0.169	−0.131	−0.161	0.510	−1.329
**8**	−0.222	−0.080	0.142	0.071	14.081	0.148	−0.115	−0.151	0.398	−1.065

**Table 3:** The binding affinity (kcal/mol) for **1**–**8** against different proteins, including DHFR and DNA gyrase with reference inhibitors Methotrexate and Novobiocin.

	ΔE	rmsd	H.B	EInt.	E_ele	LE	Ki	ΔE	rmsd	H.B	EInt.	E_ele	LE	Ki
**1**	−5.45	1.34	36.07	−16.26	−10.64	−11.83	2.47	−5.978	1.396	34.336	−13.795	−8.124	−11.829	2.471
**2**	−7.00	1.95	38.57	−22.93	−9.34	−7.24	1.98	−6.272	1.243	74.826	−21.907	−9.019	−7.242	1.980
**3**	−6.52	1.58	81.36	−18.78	−7.49	−4.67	1.54	−5.858	1.081	36.965	−15.246	−8.351	−4.666	1.540
**4**	−8.44	1.46	32.85	−25.31	−8.15	−6.14	1.81	−6.020	1.030	81.941	−18.905	−9.377	−8.654	2.158
**5**	−6.67	1.88	44.24	−17.35	−9.50	−8.65	2.16	−5.762	1.374	44.248	−21.344	−9.142	−5.655	1.733
**6**	−7.86	1.29	18.08	−24.58	−7.07	−4.19	0.94	−6.116	3.687	55.830	−18.595	−8.311	−6.928	1.936
**7**	−6.35	1.12	52.20	−22.09	−10.31	−5.66	1.73	−6.391	2.993	49.144	−14.564	−8.344	−4.769	1.562
**8**	−6.53	1.71	35.38	−14.32	−8.29	−1.17	1.94	−6.822	1.409	36.965	−18.855	−8.942	−1.168	0.155
**Ref.**	−7.85	1.68	−203.76	−27.82	−10.09	−6.58	1.88	−7.240	1.372	25.581	−21.869	−9.444	−6.138	1.815

ΔG: Free binding energy of the ligand from a given conformer, E.Int.: Affinity binding energy of hydrogen bond interaction with receptor, H.B.: Hydrogen bonding energy between protein and ligand. Eele: Electrostatic interaction with the receptor, L.E.: Ligand efficacy. Ki: inhibition constant.

**Table 4:** Inhibitory activity of imidazo[4,5-b]pyridine derivatives (**4** and **6**).

	Strains	*B. cereus*	*E. coli*
Products			
**(4)**		+	-
**(6)**		+	+

+ Presence of inhibition halo, - absence of inhibition halo.

**Table 5:** In vitro antibacterial activity of imidazo[4,5-b]pyridine derivatives (**4** and **6**).

Concentration mg/mL	4		6	
	*B. cereus*	*E. coli*	*B. cereus*	*E. coli*
**5**	-	+	-	-
**2.5**	-	+	-	-
**1.25**	-	+	-	+
**0.625**	-	+	-	+
**0.312**	-	+	-	+
**0.15**	-	+	+	+
**0.07**	-	+	+	+
**0.03**	+	+	+	+
**0.01**	+	+	+	+
**0.005**	+	+	+	+
**0.0025**	+	+	+	+

## Supplementary Materials

The following supporting information can be downloaded at: https://www.mdpi.com/article/10.3390/molecules28073197/s1, Figure S1: 1H NMR spectrum of **4**; Figure S2: 1H NMR spectrum of **6**; Figure S3: 13C NMR spectrum of **6**; Figure S4: 1H NMR spectrum of **7**; Figure S5: 1H NMR spectrum of **8**; Figure S6: 13C NMR spectrum of **8**; Figure S7: Oretp view of **3**, **6** and **8**; Table S1. Sample, Data collection and structure refinement crystal data for **3**; Table S2. Sample, Data collection and structure refinement crystal data for **6**; Table S3. Sample, crystal data, data collection and structure refinement for **8**; Table S4: the H-bond geometry (Å, °) in **3**, **6** and **8** molecules.

## Sample Availability

Sample compounds available with authors.
