# Supplementary material for: Exploring Antimicrobial Features for New Imidazo[4,5-b]pyridine Derivatives Based on Experimental and Theoretical Study
Source: Molecules. 2023 Apr 4;28(7):3197. doi: 10.3390/molecules28073197 (PMC10096078; doi:10.3390/molecules28073197)
Supplement: Supplementary file 1 [file molecules-28-03197-s001.zip › molecules-2249881-supplementary.pdf]

**Exploring antimicrobial feature for new imidazo[4,5-b] pyridine Derivatives based on experimental and theoretical study**

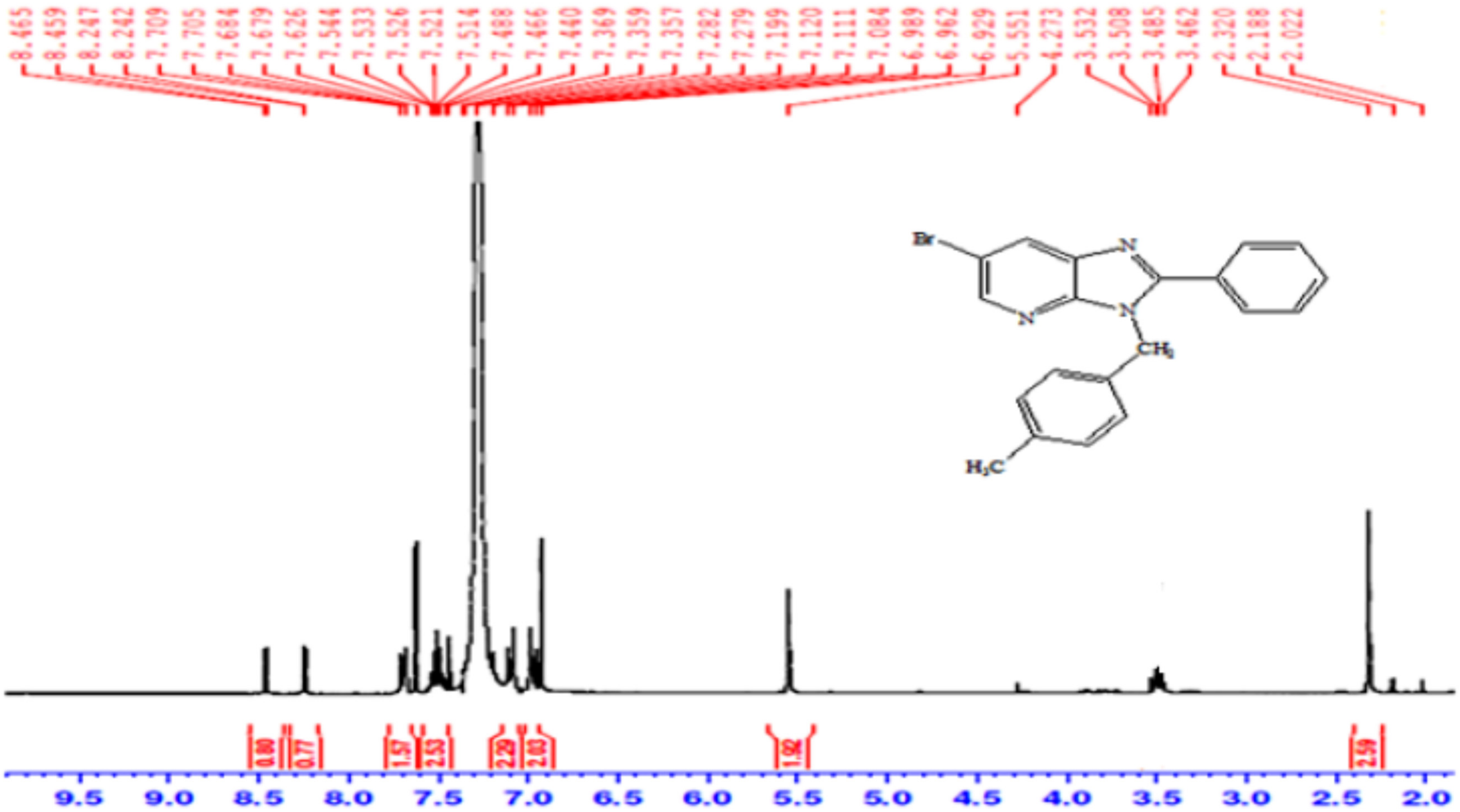


Figure S1: $^{1}$H NMR spectrum of **4**

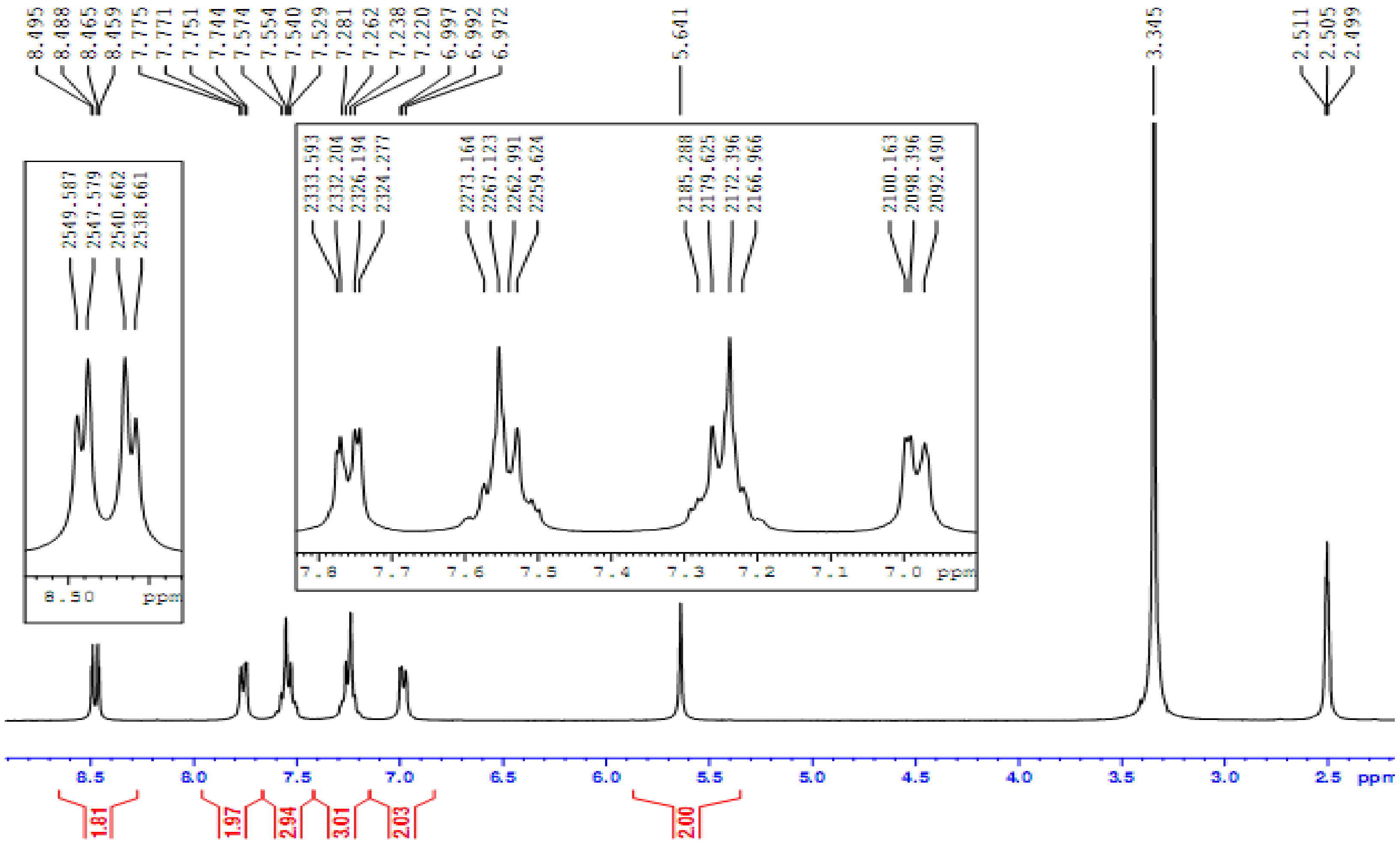


Figure S2: $^{1}$H NMR spectrum of **6**

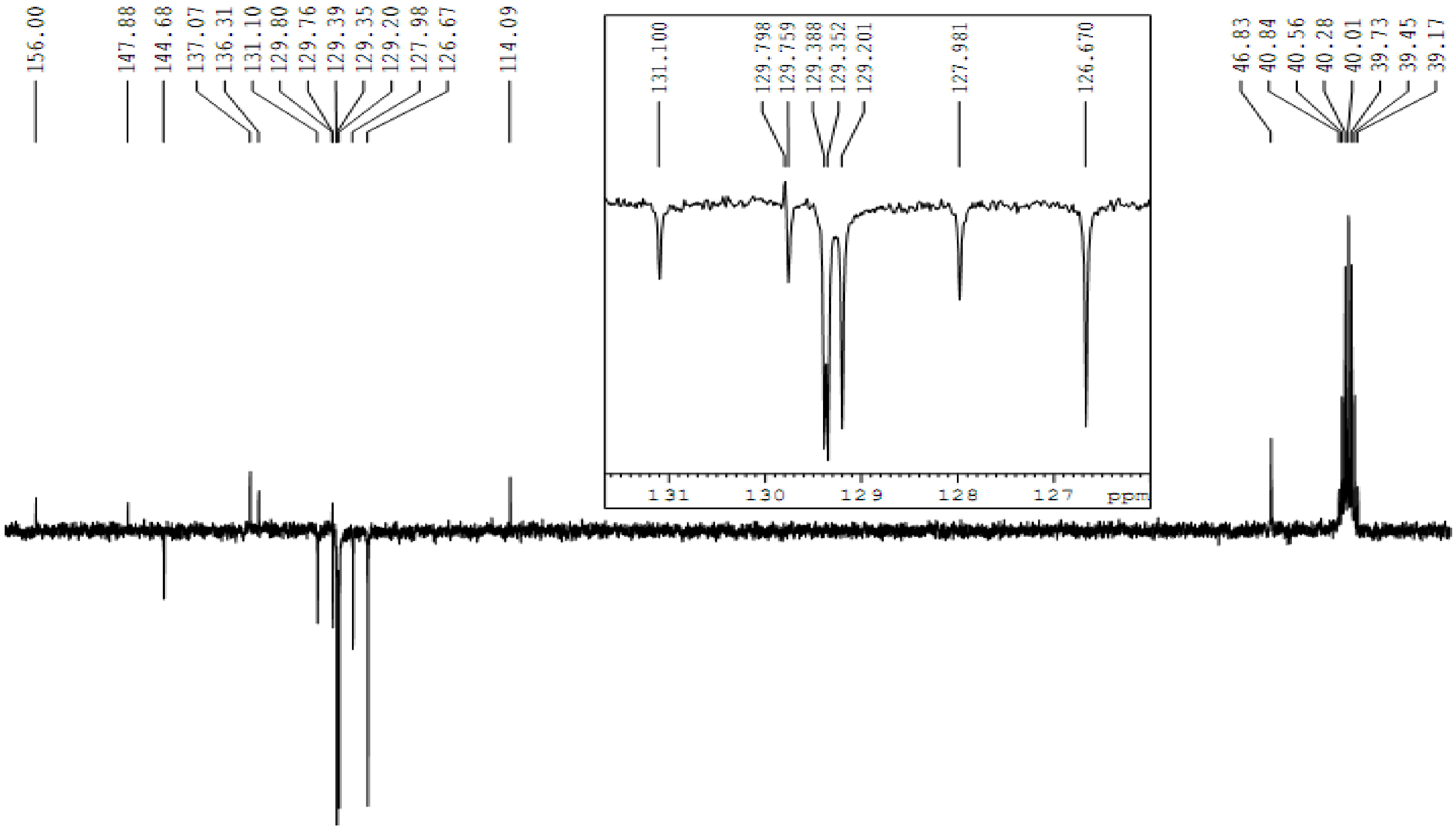


Figure S3: $^{13}C$ NMR spectrum of **6**

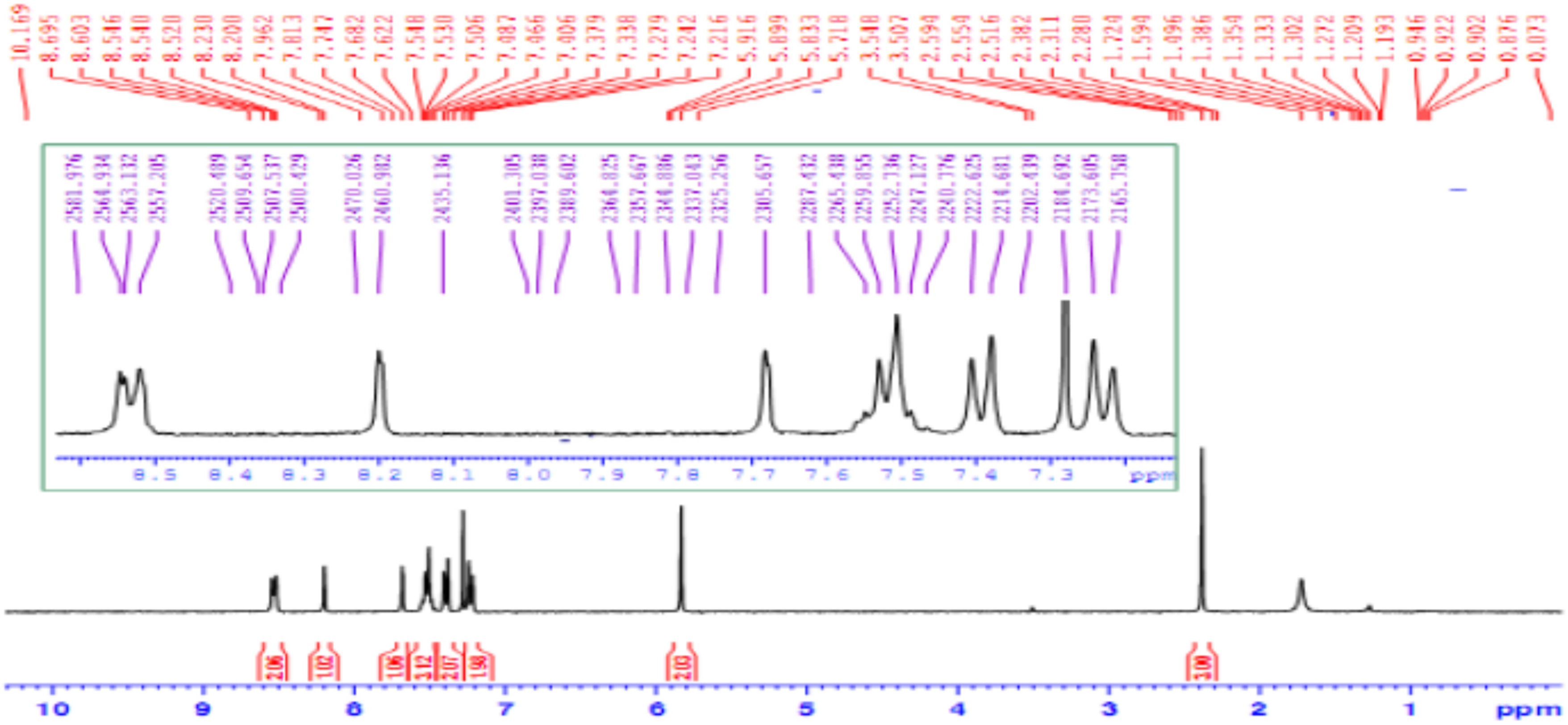

Figure S4: $^{1}$H NMR spectrum of **7**

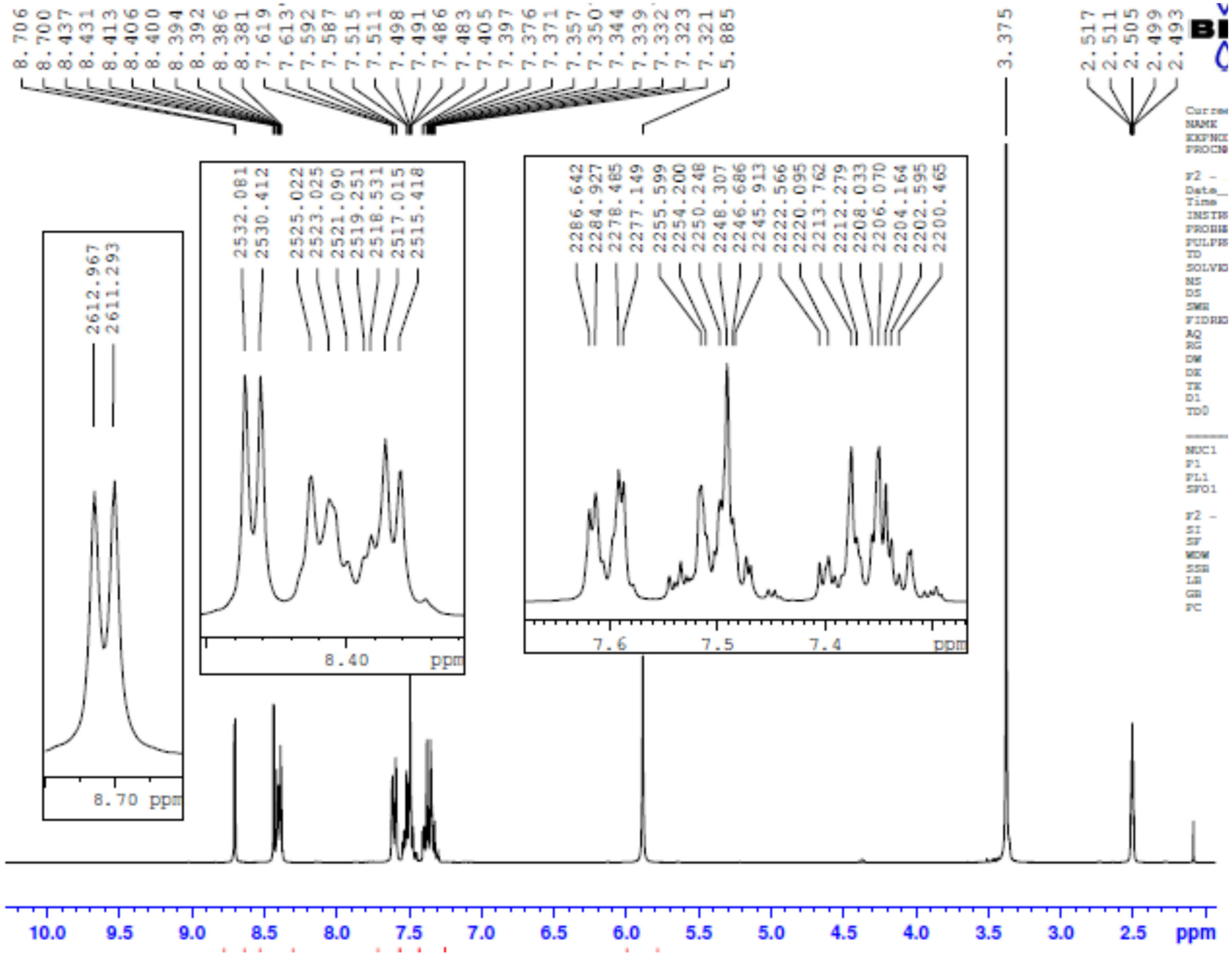

Figure S5: $^{1}$H NMR spectrum of **8**

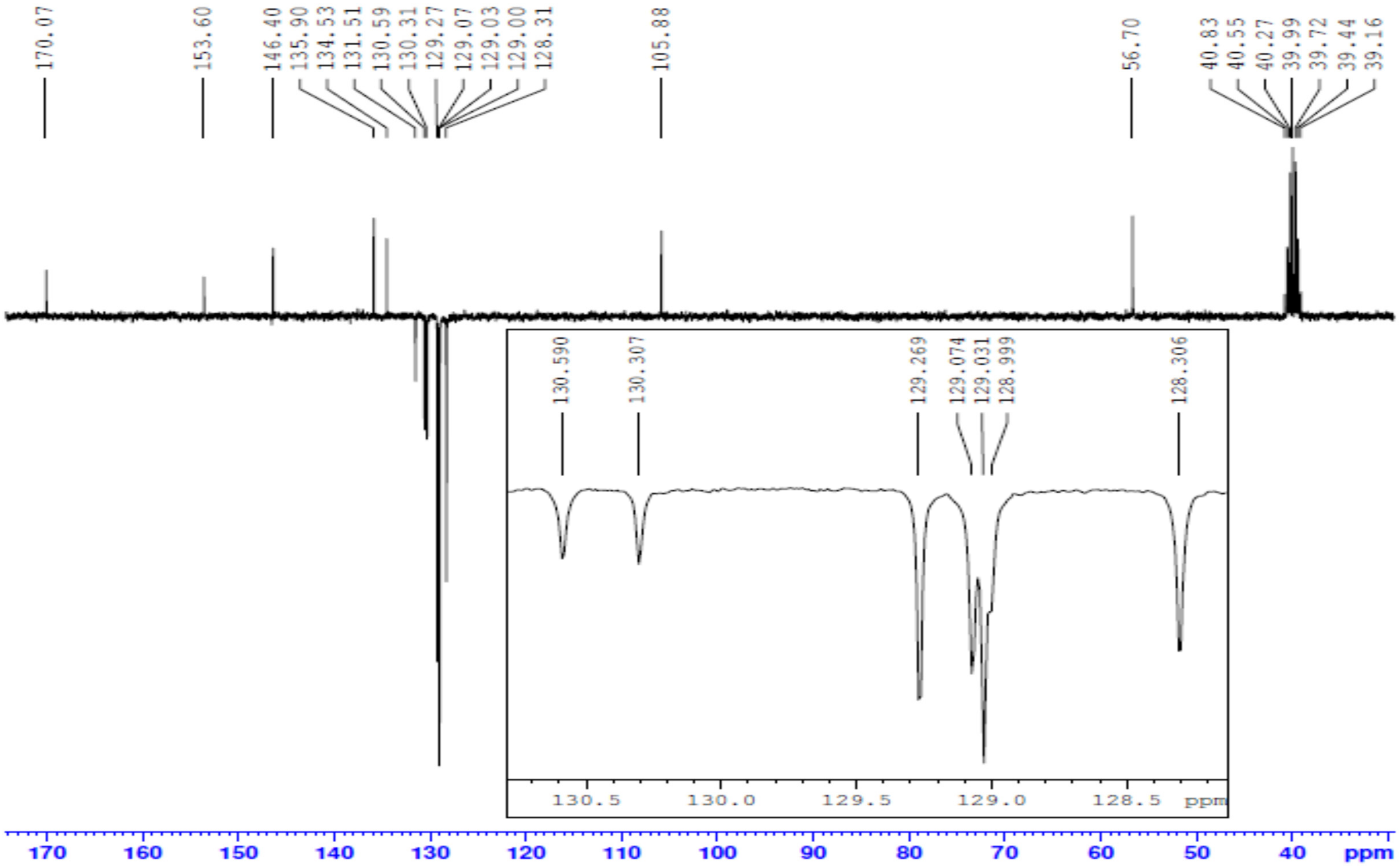


Figure S6: $^{13}C$ NMR spectrum of **8**

| 3 | 6 | 8 |
|---|---|---|
| 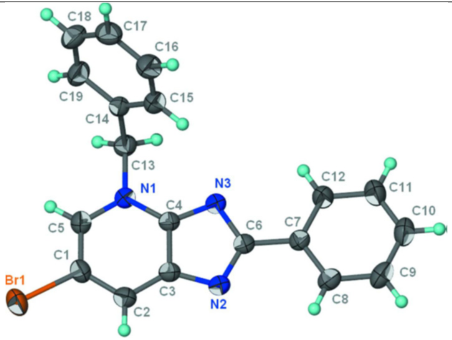 | 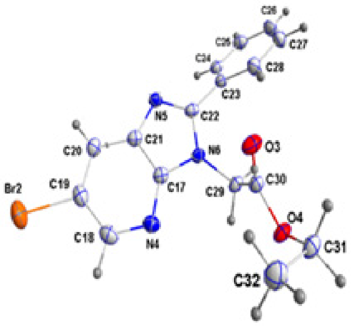 | 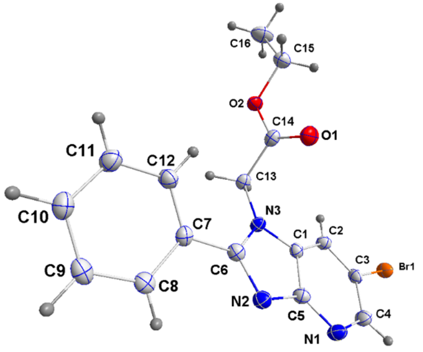 |





Figure S7: Oretp view of **3, 6 and 8**

**Table S1. Sample, Data collection and structure refinement crystal data for 3.**

| | |
|---|---|
| **C19H14BrN3** | **F(000) = 736** |
| **Mr = 364.24** | Dx = 1.530 Mg m−3 |
| **Monoclinic, P21/c** | Mo K$\alpha$ radiation, $\lambda$ = 0.71073 Å |
| **Hall symbol: -P 2ybc** | Cell parameters from 9876 reflections |
| **a = 8.6613 (6) Å** | θ = 2.4–27.2° |
| **b = 19.7631 (13) Å** | μ = 2.60 mm−1 |
| **c = 9.3683 (6) Å** | T = 293 K |
| **β = 99.647 (3)°** | Prism, brown |
| **V = 1580.93 (18) Å3** | 0.28 × 0.24 × 0.20 mm |
| **Z = 4** | |
| **Bruker X8 APEXII diffractometer** | 4613 independent reflections |
| **Radiation source: fine-focus sealed tube** | 3492 reflections with I > 2σ(I) |
| **graphite** | Rint = 0.035 |
| **φ and ω scans** | θmax = 30.1°, θmin = 2.4° |
| **Absorption correction: multi-scan (SADABS; Sheldrick, 1996)** | h = −12→11 |
| **Tmin = 0.529, Tmax = 0.624** | k = −27→27 |
| **57936 measured reflections** | l = −13→13 |
| **Refinement on $F^2$** | Primary atom site location: structure invariant direct methods |
| **Least-squares matrix: full** | Secondary atom site location: difference Fourier map |

| | |
|---|---|
| **R[F2> 2σ(F2)] = 0.032** | Hydrogen site location: inferred from neighbouring sites |
| **wR(F2) = 0.098** | H-atom parameters constrained |
| **S = 1.00** | w = 1/[σ2(Fo2) + (0.0518P)2+ 0.5269P]<br>where P = (Fo2 + 2Fc2)/3 |
| **4613 reflections** | (Δ/σ)max = 0.001 |
| **208 parameters** | Δϱmax = 0.63 e $Å^{-3}$ |
| **0 restraints** | Δϱmin = −0.51 e $Å^{-3}$ |

**Table S2. Sample, Data collection and structure refinement crystal data for 6.**

| | | |
|---|---|---|
| **Chemical formula** | $\mathbf{C_{16}H_{14}BrN_3O_2}$ | |
| **Formula weight** | 360.21 g/mol | |
| **Temperature** | 150(2) K | |
| **Wavelength** | 0.71073 Å | |
| **Crystal size** | 0.156 x 0.244 x 0.364 mm | |
| **Crystal habit** | colorless block | |
| **Crystal system** | monoclinic | |
| **Space group** | P 1 21/c 1 | |
| **Unit cell dimensions** | a = 21.1444(14) Å | $\alpha$ = 90° |
| | b = 7.6970(5) Å | $\beta$ = 118.0730(10)° |
| | c = 21.2671(14) Å | $\gamma$ = 90° |
| **Volume** | 3054.0(3) $Å^3$ | |
| **Z** | 8 | |
| **Density (calculated)** | 1.567 $g/cm^3$ | |
| **Absorption coefficient** | 2.702 $mm^{-1}$ | |
| **F(000)** | 1456 | |
| **Diffractometer** | Bruker Smart APEX CCD | |
| **Radiation source** | fine-focus sealed tube, MoK$\alpha$ | |
| **Theta range for data collection** | 1.92 to 28.80° | |
| **Index ranges** | -28<=h<=28, -10<=k<=10, -28<=l<=28 | |
| **Reflections collected** | 56392 | |
| **Independent reflections** | 7958 [R(int) = 0.0369] | |
| **Coverage of independent reflections** | 99.6% | |
| **Absorption correction** | numerical | |
| **Max. and min. transmission** | 0.6780 and 0.4400 | |
| **Structure solution technique** | direct methods | |
| **Structure solution program** | *SHELXT* (Sheldrick, 2015) | |

| | | |
|---|---|---|
| **Refinement method** | Full-matrix least-squares on $F^2$ | |
| **Refinement program** | SHELXL-2014/7 (Sheldrick, 2014) | |
| **Function minimized** | $\Sigma$ w($F_o^2$ - $F_c^2$)$^2$ | |
| **Data / restraints / parameters** | 7958 / 0 / 399 | |
| **Goodness-of-fit on $F^2$** | 1.035 | |
| **$\Delta/\sigma_{max}$** | 0.002 | |
| **Final R indices** | 6411 data; I>2$\sigma$(I) | R1 = 0.0307, wR2 = 0.0755 |
| | all data | R1 = 0.0430, wR2 = 0.0803 |
| **Weighting scheme** | w=1/[$\sigma^2$($F_o^2$)+(0.0421P)$^2$+0.9304P]<br>where P=($F_o^2$+2$F_c^2$)/3 | |
| **Largest diff. peak and hole** | 0.782 and -0.639 eÅ$^{-3}$ | |
| **R.M.S. deviation from mean** | 0.063 eÅ$^{-3}$ | |

**Table S3. Sample, crystal data, data collection and structure refinement for 8**

| | | |
|---|---|---|
| **Chemical formula** | $\mathbf{C_{16}H_{14}BrN_3O_2}$ | |
| **Formula weight** | 360.21 g/mol | |
| **Temperature** | 150(2) K | |
| **Wavelength** | 0.71073 Å | |
| **Crystal size** | 0.060 x 0.150 x 0.210 mm | |
| **Crystal habit** | colorless plate | |
| **Crystal system** | monoclinic | |
| **Space group** | P 1 21/n 1 | |
| **Unit cell dimensions** | a = 14.6923(8) Å | $\alpha$ = 90° |
| | b = 6.1988(3) Å | $\beta$ = 92.9110(10)° |
| | c = 16.2254(8) Å | $\gamma$ = 90° |
| **Volume** | 1475.82(13) $Å^3$ | |
| **Z** | 4 | |
| **Density (calculated)** | 1.621 $g/cm^3$ | |
| **Absorption coefficient** | 2.796 $mm^{-1}$ | |
| **F(000)** | 728 | |
| **Diffractometer** | Bruker Smart APEX CCD | |
| **Radiation source** | fine-focus sealed tube, MoK$\alpha$ | |
| **Theta range for data collection** | 1.82 to 29.18° | |
| **Index ranges** | -20<=h<=20, -8<=k<=8, -22<=l<=21 | |
| **Reflections collected** | 27419 | |
| **Independent reflections** | 3981 [R(int) = 0.0436] | |
| **Coverage of independent reflections** | 99.7% | |
| **Absorption correction** | multi-scan | |
| **Max. and min. transmission** | 0.8500 and 0.5910 | |
| **Structure solution technique** | direct methods | |
| **Structure solution program** | *SHELXT* (Sheldrick, 2015) | |

| | | |
|---|---|---|
| **Refinement method** | Full-matrix least-squares on $F^2$ | |
| **Refinement program** | SHELXL-2014/7 (Sheldrick, 2014) | |
| **Function minimized** | $\Sigma w(F_o^2 - F_c^2)^2$ | |
| **Data / restraints / parameters** | 3981 / 0 / 200 | |
| **Goodness-of-fit on $F^2$** | 1.064 | |
| **$\Delta/\sigma_{max}$** | 0.001 | |
| **Final R indices** | 3236 data; $I>2\sigma(I)$ | R1 = 0.0307, wR2 = 0.0781 |
| | all data | R1 = 0.0414, wR2 = 0.0814 |
| **Weighting scheme** | $w=1/[\sigma^2(F_o^2)+(0.0468P)^2]$<br>where $P=(F_o^2+2F_c^2)/3$ | |
| **Largest diff. peak and hole** | 0.758 and -0.349 eÅ$^{-3}$ | |
| **R.M.S. deviation from mean** | 0.079 eÅ$^{-3}$ | |

Table S4: the H-bond geometry (Å, °) in **3,6 and 8** molecules.

| | | | | |
|---|---|---|---|---|
| | | **3** | | |
| ***D*—H *A*** | *D*—H | H *A* | D *A* | *D*—H *A* |
| **H24— O1H15B** [i] | 0.117 | 2.603 | 2.939 | 110 |
| **H28—O3H31 A**[ii] | 0.136 | 2.652 | 2.311 | 119 |
| **C13—N5 H13 A**[i] | 0.546 | 2.652 | 3.165 | 135.5 |
| Symmetry codes: (i) −*x*+1, *y*+1/2, −*z*+3/2; (ii) *x*, −*y*+1/2, *z*−1/2. | | | | |
| | | **6** | | |
| ***D*—H *A*** | *D*—H | H *A* | D *A* | *D*—H *A* |
| **C14—O1 H16A**[i] | 0.119 | 2.601 | 1.206 | 124 |
| **C5—N2 H13A**[ii] | 0.440 | 2.310 | 1.338 | 125 |
| **C4—N1H4**[i] | 0.150 | 2.600 | 1.331 | 118.2 |
| Symmetry codes: (i) x,-1+y,z (ii) *1-x,-y,1-z*. | | | | |
| | | **8** | | |
| ***D*—H *A*** | *D*—H | H *A* | D *A* | *D*—H *A* |
| **C13—C14 H13A**[i] | 0.252 | 2.148 | 1.385 | 109.3 |
| **C15—C16 H10B**[ii] | 0.95 | 1.370 | 1.370 | 109.6 |
| Symmetry codes: (i) 1-x,1-y,1-z (ii) 1/2-x,-1/2+y,1/2-z | | | | |
